# Relationships of beans intake with chronic kidney disease in rural adults: A large-scale cross-sectional study

**DOI:** 10.3389/fnut.2023.1117517

**Published:** 2023-04-04

**Authors:** Lei Yin, Xiaokang Dong, Wei Liao, Xiaotian Liu, Zhaohui Zheng, Dongwei Liu, Chongjian Wang, Zhangsuo Liu

**Affiliations:** ^1^Department of Nephrology, The First Affiliated Hospital of Zhengzhou University, Zhengzhou, Henan, China; ^2^Department of Epidemiology and Biostatistics, College of Public Health, Zhengzhou University, Zhengzhou, Henan, China; ^3^Department of Rheumatology, The First Affiliated Hospital of Zhengzhou University, Zhengzhou, China

**Keywords:** beans intake, chronic kidney disease, albuminuria, kidney injury, rural adults

## Abstract

**Background and aims:**

Dietary factors play an important role in the development of chronic kidney disease (CKD). However, evidence on the relationship of beans consumption with CKD remains limited and inconclusive, especially in the middle-and low-income populations. The current study aimed to investigate the relationships of beans intake with indicators of kidney injury and CKD prevalence in rural adults.

**Methods:**

A total of 20,733 rural adults from the Henan Rural Cohort Study in 2018–2022 were included. The total beans intake was collected using a validated food frequency questionnaire. Indicators of kidney injury and CKD was determined by the estimated glomerular filtration rate and the urinary albumin to creatinine ratio. Generalized linear regression and logistic regression models were applied to estimate the relationship of beans intake with continuous and dichotomized indicators of renal function, respectively.

**Results:**

Of the 20,733 participants, 2,676 (12.91%) subjects were identified as CKD patients. After adjusting for potential confounders, participants in the higher quartiles of beans intake had a lower prevalence of CKD (odds ratio and 95% confidence interval, *OR* (95%*CI*); Q2: 0.968(0.866–1.082); Q3: 0.836(0.744–0.939); Q4: 0.854(0.751–0.970)) and albuminuria (Q2: 0.982(0.875–1.102); Q3: 0.846(0.750–0.954); Q4: 0.852 (0.746–0.973)), compared with the Q1. Per 50 g/day increment in beans intake was significantly associated with a 5 and 4% decreased prevalence of albuminuria and CKD, respectively. These inverse relationships were also significant in the subgroups of men, elder, and high-income participants (*p* < 0.05).

**Conclusion:**

Dietary beans intake was inversely associated with the prevalence of albuminuria and CKD in rural adults, suggesting that promoting soy food intake might help reduce the occurrence of CKD in rural adults.

## Introduction

Chronic Kidney Disease (CKD), a chronic disease characterized by persistent abnormalities in renal structure and function, has been a global public health issue with a high mortality risk and severer disease burden in the past decades (1, 2). There were over 697 million CKD patients worldwide in 2019, according to the GBD 2019 Diseases and Injuries Collaborators (3). The prevalence of CKD is high and is increasing rapidly, especially in developing countries such as China, which has the largest number of CKD patients globally, with 132 million in 2019 (about a prevalence of 10%) (4). Previous studies have reported many established risk factors of CKD, including hypertension, diabetes, dyslipidemia, metabolic syndrome, hyperuricemia, and lifestyle factors such as socioeconomic status, physical activity, alcohol consumption and so on (5–8). However, evidence on the relationship between various dietary factors and the development of CKD is mixed and warrants further investigation.

Soy is a popular food and a valuable source of nutrients, phytochemicals, and bioactive compounds (9). In Asian countries such as China, Japan, and Korea, soy and its products have been consumed for centuries (10). The components of soy food have multiple health benefits. Many studies have shown that soy or beans intake is associated with a lower prevalence of hypertension, diabetes, hyperuricemia, obesity and CVD (10–12), which are closely related to the development of CKD. However, the relationships between soy food consumption and renal function and CKD remain controversial due to limited evidence. Physicians recommend soy products for CKD patients as they are a high-quality plant-based protein source and other nutrients (13). Still, many people did not consume soy foods due to concerns about adverse effects on the progress of CKD (14). Emerging evidence from animal and clinical studies demonstrated that soy protein consumption might delay the development of renal dysfunction and CKD (15, 16). However, some studies did not observe a beneficial effect of bean intake on CKD. They found that soy protein intake did not significantly alter glomerular filtration rate or attenuate proteinuria (17, 18). Furthermore, one observational study showed that replacing animal protein with soy increased urinary protein excretion (19). In addition, a recent population-based nutritional study found that adherence to a healthy dietary pattern, including soy products, is associated with a lower risk of CKD progression (20). However, few studies evaluated the independent health effects of soy or beans consumption on CKD or kidney function, especially in large-sample epidemiological surveys.

Among the rural population, beans and soybean products are more common compared to the animal-based dietary pattern of the urban population (21). Moreover, the prevalence of CKD has been as high as 16.40% in recent years (8). Given the mixed findings of beans intake in the management of CKD, therefore, we examined the relationships between total beans intake and kidney injury indicators and CKD prevalence based on the latest data from the Henan Rural Cohort Study. Furthermore, subgroup analyses were conducted to explore effect modifiers of the relationships of total beans intake and several indicators of kidney injury.

## Materials and methods

### Study population

The study population was derived from the Henan Rural Cohort Study (Registration number: ChiCTR-OOC-15006699), which has been conducted between July 2018 to September 2021. A total of 28,628 rural residents aged 18–79 completed the questionnaires and physical examination in the follow-up study in 2018–2022. Of these participants, 26,030 were interviewed at baseline and 2,598 were new to the cohort. The detailed study design, data collection, and measurements have been described elsewhere (22). In the current study, 21,079 participants completed the routine blood and urine measurements. Furthermore, 211 participants were excluded due to a diagnosis of cancer (*N* = 200) or kidney failure (*N* = 11); 135 participants with missing data on beans intake (*N* = 15), eGFR (*N* = 4) and ACR (*N* = 116) were further excluded, leaving 20, 733 participants for the current analysis in final. The flowchart of participant inclusion is shown in Supplementary Figure S1. The study protocols were approved by the Zhengzhou University Life Science Ethics Committee. All participants gave informed consent before the investigation.

### Dietary assessment and beans intake

The current study used a semi-quantitative 13-item food frequency questionnaire (FFQ) to collect dietary information. Individual data for the FFQ were obtained by experienced interviewers during a face-to-face visit. The 13-item FFQ included staple foods, red meat, white meat, fish, eggs, milk, fruits, vegetables, beans, nuts, pickles, cereal, and animal oil. Participants were asked to report the frequency (never, day, week, month, year) and the amount (kilograms, grams) of their consumption in the past 12 months. A validation study has demonstrated the reliability of the FFQ (23). Detailed information about the calculation of various types of food has been described in our previous study (23). For example, various types of food groups, including staple food such as rice, noodles and steamed bread; red meat such as pork, lamb and beef; white meat such as chicken and duck; fish including freshwater and marine fish; eggs such as chicken, duck and goose eggs; milk and dairy products including milk, goat’s milk, yogurt, cheese, and other dairy products; fruits; vegetables; nuts and peanuts; grains like corn, sweet potatoes and sorghum. In particular, beans intake in this study was identified as the total consumption of all beans and products commonly eaten by local people, such as soybean, lentil, red bean, mung bean, black bean, pea, tofu, soy milk, dried tofu, vegetarian chicken, etc. (21). Furthermore, all participants were divided into four groups according to quartiles of daily total beans intake in the main analyses: Q1, beans intake <6.67 g/d; Q2, beans intake =6.68–16.67 g/d; Q3, beans intake =16.68–50 g/d; and Q4, beans intake >50 g/d.

### Outcome assessment

After an overnight fast of at least 8 hours, all participants were recruited to the health examination center in the local communities in the morning. Blood samples were taken from the antecubital vein by well-trained nurses and stored them under the right conditions. Serum samples were forwarded to measure biochemical indicators including fasting blood glucose, insulin, total cholesterol, triglycerides, high-density lipoprotein, low-density lipoprotein, serum uric acid and serum creatinine (OCHE Cobas C501 automatic biochemical analyzer). The eGFR was calculated using the 2009 CKD Epidemiology Collaboration equation (CKD-EPI creatinine equation) (23). Urine samples were also collected to detect routine urine substances such as urinary albumin and urine creatinine. The urinary albumin to creatinine ratio (ACR, mg/g) was calculated.

Albuminuria was defined as participants with an ACR > 30 mg/g. Indicators of kidney injury were the presence of a reduced eGFR (eGFR <60 ml/min/1.73 m^2^) or albuminuria. The CKD was defined as either presence of albuminuria or an estimated glomerular filtration rate (eGFR) ≤ 60 ml/min per 1.73 m^2^ (8, 24).

### Potential covariates

A validated questionnaire was used for potential covariates to collect basic characteristics such as age, gender, education level, average monthly income, smoking status, drinking status, and meat and diet intake, as previously reported (21). Education level was classified into ≤primary, middle, and ≥ senior high school. Averaged monthly income was divided into three groups (<500 RMB, 500–1,000 RMB and ≥ 1,000 RMB groups). Smoking and drinking status were split into current or other groups. Those who had current smoked for half a year or more and one cigarette per day or more were defined as current smokers. Participants who consumed alcohol 12 or more times yearly without abstinence were considered current drinkers. Physical activity was classified into light, moderate, and vigorous levels according to the International Physical Activity Questionnaire (IPAQ) (25). Body mass index (BMI) was calculated according to the measurements of height and weight of participants. Especially, according to the FFQ, daily intakes of red meat (g/day), white meat (g/day), fish (g/day), egg (g/day), milk (g/day), vegetable (g/d) and fruit (g/d) were selected as potential covariates which were related with CKD (19). In addition, several chronic disease conditions (hypertension, T2DM, dyslipidemia and hyperuricemia) were objectively assessed and determined as previous definitions (26).

### Statistical analysis

The basic characteristics of study participants are presented as the mean (standard deviation, SD) for continuous variables and the number (percentage) for categorical variables. According to the CKD condition (Yes or No), basic characteristics were compared by using Student’s *t*-test and Chi-square test for continuous and categorical variables, respectively. Participants were divided into quartiles of total beans intake (Q1-Q4). Then, the crude prevalence of reduced eGFR (eGFR <60 mL/min/1.73 m^2^), albuminuria and CKD were calculated and compared according to quartiles of total beans intake.

In the main analysis, the relationships of total beans intake with eGFR and ACR (continuous outcomes) in the total population were first analyzed by generalized linear regression models. Effect estimates are presented as regression coefficients (***β***) and corresponding 95% confidence intervals (*CIs*). Moreover, a multivariable-adjusted logistic regression model was used to estimate the odds ratios (*ORs*) and 95% *CIs* of the relationships between quartiles of total beans intake and the prevalence of reduced eGFR, albuminuria and CKD (the dichotomous outcomes). All regression models were analyzed using the lowest quartiles group as the reference group. Tests for *p* for linear trend were conducted across quartiles using the median beans intake in each quartile as a linear variable in the regression models. Two models were shown: Model 1 was adjusted for age, gender, education level, averaged monthly income, current smoker, current drinker, physical activity and BMI; Model 2 was adjusted for age, gender, education level, averaged monthly income, current smoker, current drinker, physical activity, BMI, red-meat (g/day), white-meat (g/day), fish (g/day), egg (g/day), milk (g/day), vegetable (g/d) and fruit (g/d).

Sensitivity analyses were conducted to test the robustness of our estimates: first, we repeated the analyses to evaluate the effects of quartiles of total beans intake on reduced eGFR, albuminuria and CKD prevalence by additionally adjusting for T2DM, hypertension, dyslipidemia and hyperuricemia conditions based on model 2. Second, we divided the total population into men and women, calculated the soy quartiles by gender, and assessed the respective effects in the regression analyses. In addition, given that dietary pattern is more important than single food item intake. We also have adjusted for dietary patterns in place of single food items in model 2 to test the robustness of total beans intake on reduced eGFR, albuminuria and CKD prevalence. The four-cluster dietary patterns were obtained by factor analysis using the standard principal component analysis method referring to previous studies (27, 28).

Subgroup analyses were performed to evaluate the effects of per 50 g/day increment in total beans intake on reduced eGFR, albuminuria and CKD prevalence by several factors: age (<65 or ≥ 65), sex (men or women), education level (≤primary school, middle school or ≥ senior high school) and averaged monthly income (< 500 RMB, 500–1,000 RMB or ≥ 1,000 RMB). Effect modification analysis was performed by adding an interaction term between total beans intake and the testing variable included in the regression model. The interactions were considered present if a cross-product term was statistically significant.

All data were analyzed using SPSS software version 21.0 and R software version 3.5.3. Two-tailed *p* values <0.05 were considered statistically significant.

## Results

### Characteristics of participants

The demographic characteristics of the individuals according to their CKD status, are shown in Table 1. Among 20,733 participants, 2,676 (12.91%) subjects with CKD. The mean age of the study population was 60.19 years, and 12,908 (39.6%) of the participants were women. Compared with non-CKD participants, participants with CKD were associated with older age, lower education level, average monthly income and physical activity level; lower intake of red meat, fish, milk, fruit, beans; and lower eGFR (all *p* < 0.05); while they had higher BMI, higher ACR, hypertension, T2DM, dyslipidemia and hyperuricemia (all *p* < 0.001). Furthermore, similar distributions of demographic characteristics according to indicators of kidney injury were presented in Supplementary Table S1 in Supplementary materials. There were 366 (1.77%) participants with reduced eGFR (eGFR<60 mL/min/1.73 m^2^) and 2,448 (11.80%) participants with albuminuria.

**Table 1:** Basic characteristics of study participants with and without CKD (*n* = 20,733).

Variables	Total (*n* = 20,733)	CKD		*p*-value
		No (*n* = 18,057)	Yes (*n* = 2,676)	
Age (year, mean ± SD)	60.19 ± 11.34	59.62 ± 11.30	64.00 ± 10.85	0.021
Gender (*n*, %)				0.005
Men	7825 (37.74)	6881 (38.11)	944 (35.28)	
Women	12908 (62.26)	11176 (61.89)	1732 (64.72)	
Education level (*n*, %)				<0.001
≤Primary school	10152 (48.97)	8561 (47.50)	1591 (59.54)	
Middle school	7915 (38.18)	7055 (39.14)	860 (32.19)	
≥Senior high school	2628 (12.68)	2407 (13.36)	221 (8.27)	
Average monthly income (*n*, %)				<0.001
<500 RMB	7112 (34.30)	5978 (33.11)	1134 (42.38)	
500–1,000 RMB	5520 (26.62)	4853 (26.88)	667 (24.93)	
>1,000 RMB	8101 (39.07)	7226 (40.01)	875 (32.70)	
Current smoker, *n* (%)	3418 (16.49)	3052 (16.90)	366 (13.68)	<0.001
Current drinker, *n* (%)	3094 (14.92)	2775 (15.36)	319 (11.92)	<0.001
Physical activity, *n* (%)				<0.001
Light	8379 (40.41)	7120 (39.43)	1259 (47.05)	
Moderate	6972 (33.63)	6113 (33.85)	859 (32.10)	
Vigorous	5382 (25.96)	4824 (26.72)	558 (20.85)	
BMI (kg/m^2^), mean ± SD	24.59 ± 3.50	24.50 ± 3.45	25.15 ± 3.75	<0.001
Red-meat (g/day), mean ± SD	37.81 ± 72.81	38.59 ± 74.74	32.60 ± 57.85	0.003
White-meat (g/day), mean ± SD	21.06 ± 58.36	21.23 ± 54.65	19.90 ± 78.95	0.580
Fish (g/day), mean ± SD	7.71 ± 32.12	7.91 ± 32.35	6.36 ± 30.48	0.001
Egg (g/day), mean ± SD	48.07 ± 40.40	48.27 ± 40.51	46.70 ± 39.65	0.205
Milk (g/day), mean ± SD	49.23 ± 91.81	48.57 ± 90.56	53.73 ± 99.70	<0.001
Vegetable intake (g/d), mean ± SD	368.38 ± 223.09	368.65 ± 221.86	366.54 ± 231.30	0.179
Fruit intake (g/d), mean ± SD	147.88 ± 167.41	150.59 ± 170.11	129.61 ± 146.63	0.016
Beans intake (g/day), mean ± SD	42.15 ± 80.26	42.82 ± 81.65	37.67 ± 69.97	0.006
Hypertension, *n* (%)	6443 (31.08)	4,946 (27.40)	1,497 (55.94)	<0.001
T2DM, *n* (%)	2813 (13.57)	2094 (11.61)	719 (26.95)	<0.001
Dyslipidemia, *n* (%)	12525 (60.41)	10697 (59.26)	1828 (68.34)	<0.001
Hyperuricemia, *n* (%)	3163 (15.26)	2254 (14.14)	609 (22.76)	<0.001
eGFR (mL/min/1.73 m^2^), mean ± SD	101.26 ± 18.96	102.44 ± 17.30	93.27 ± 26.29	<0.001
ACR (mg/g), mean ± SD	23.35 ± 101.38	9.18 ± 6.21	118.95 ± 262.49	<0.001

CKD, chronic kidney disease; SD, standard deviation; BMI, body mass index; eGFR, estimated glomerular filtration rate; ACR, urinary albumin to creatinine ratio.

### Distributions and prevalence of kidney injury indicators by quartiles of total beans intake

**Figure 1:**
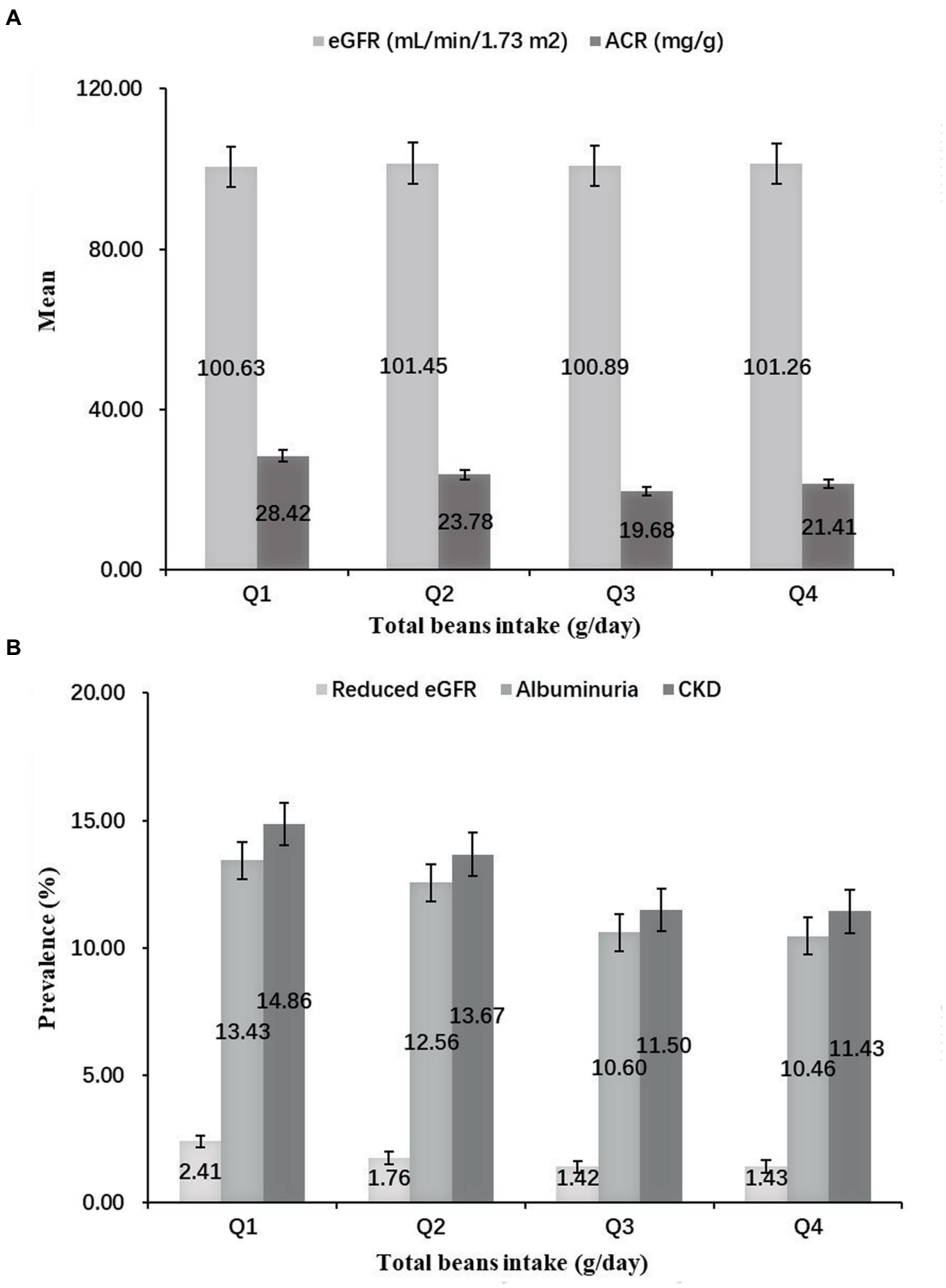
Distributions and prevalence of kidney injury indicators according to quartiles of total beans intake. **(A)** Distributions of kidney injury indicators according to quartiles of total beans intake. **(B)** Prevalence of kidney injury indicators according to quartiles of total beans intake.

Figure 1 shows a significant difference in distributions and prevalence of kidney injury indicators by quartiles of total beans intake (all *p* < 0.001). Compared with the lowest quartile (Q1), the mean levels of eGFR in Q2-Q4 were higher, but the mean levels of ACR were lower (Figure 1A). Moreover, the prevalence of reduced eGFR, albuminuria and CKD tended to be lower in higher quartile groups. In particular, the prevalence of CKD in the Q1-Q4 groups was 14.86, 13.67, 11.50, and 11.43%, respectively (Figure 1B).

### Relationship of total beans intake with kidney injury indicators and CKD prevalence

**Figure 2:**
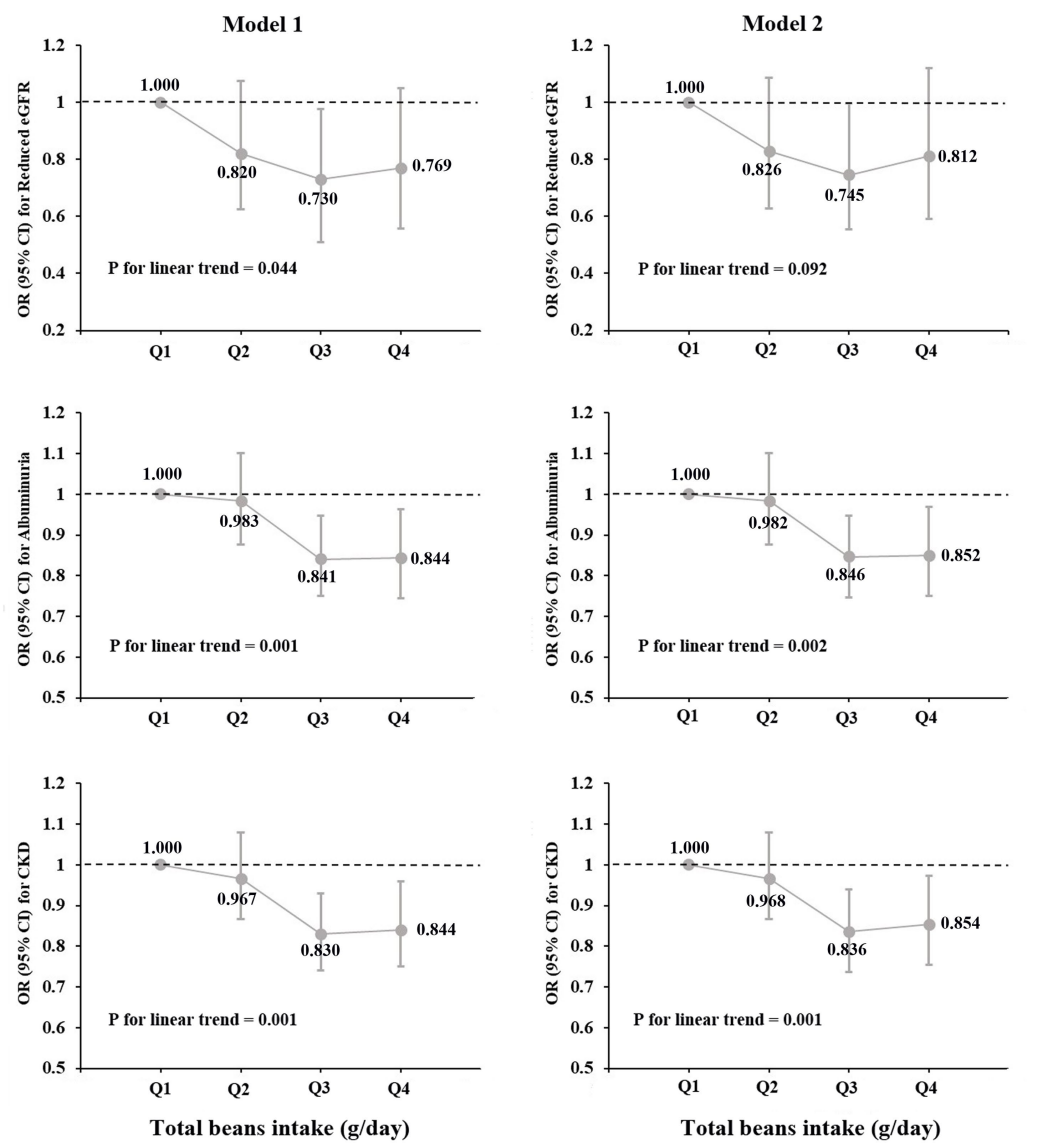
Prevalence of reduced eGFR, albuminuria and CKD by quartiles of total beans intake (*OR* and 95% *CI*).

Table 2 shows the relationships of total beans intake with eGFR and ACR levels in multivariable-adjusted regression models. In model 2, more ACR decline was observed for those in the highest quintiles of total beans intake (Q4: −6.292 mg/g; 95% *CI*, −10.46 to-2.121; *P* for linear trend <0.001) compared with those in Q1. While more eGFR decline was also observed for those in the highest quintiles (Q4: −1.526 ml/min/1.73 m2; 95% *CI*, −2.195 to-0.857; *P* for linear trend <0.001). Furthermore, when the continuous outcome variables were classified according to clinical diagnostic criteria, we found that elevated total beans intake was significantly associated with decreased prevalence of CKD and albuminuria but not with a prevalence of reduced eGFR in all models (Table 3). In the fully adjusted model, participants in the higher quartiles of beans intake had a lower prevalence of CKD (Q2: *OR*, 0.968; 95%*CI*, 0.866–1.082; Q3: *OR*, 0.836; 95%*CI*, 0.744–0.939; Q4: *OR*, 0.854; 95%*CI*, 0.751–0.970) and albuminuria (Q2: *OR*, 0.982; 95%*CI*, 0.875–1.102; Q3: *OR*, 0.846; 95%*CI*, 0.750–0.954; Q4: *OR*, 0.852; 95% *CI*, 0.746–0.973), compared with participants in the Q1 (Table 3). In addition, in Figure 2, the prevalence of albuminuria and CKD decreased with increasing quartile group (all *p* for linear trend <0.05).

**Table 2:** Multivariable-adjusted *β*-coefficients and 95% *CI* for eGFR and ACR according to quartiles of total beans intake.

Variables	Total beans intake (g/day)				*p* for linear trend
	Q1	Q2	Q3	Q4	
*eGFR (mL/min/1.73 m^2^)*					
Model 1	0 (Reference)	−0.048 (−0.663, 0.568)	−1.727 (−2.346, −1.108)	−0.537 (−1.194, 0.120)	0.001
Model 2	0 (Reference)	−0.164 (−0.775, 0.447)	−1.906 (−2.521, −1.291)	−1.526 (−2.195, −0.857)	<0.001
*ACR (mg/g)*					
Model 1	0 (Reference)	−3.695 (−7.488, 0.099)	−7.631 (−11.45, −3.815)	−5.856 (−9.906, −1.805)	<0.001
Model 2	0 (Reference)	−3.743 (−7.552, 0.065)	−7.622 (−11.46, −3.786)	−6.292 (−10.46, −2.121)	<0.001

Model 1: Adjusted for age, gender, education level, averaged monthly income, current smoker, current drinker, physical activity and body mass index.

Model 2: Adjusted for age, gender, education level, averaged monthly income, current smoker, current drinker, physical activity, body mass index, red meat (g/day), white meat (g/day), fish (g/day), egg (g/day), milk (g/day), vegetable (g/d) and fruit (g/d).

**Table 3:** Multivariable-adjusted *OR* and 95% *CI* for reduced eGFR, albuminuria and CKD according to quartiles of total beans intake.

Outcome	Total beans intake (g/day)				*p* for linear trend
	Q1	Q2	Q3	Q4	
*Reduced eGFR*					
Model 1	1 (Reference)	0.820 (0.624, 1.077)	0.730 (0.545, 0.976)	0.769 (0.563, 1.051)	0.044
Model 2	1 (Reference)	0.826 (0.628, 1.086)	0.745 (0.557, 1.000)	0.812 (0.588, 1.120)	0.092
*Albuminuria*					
Model 1	1 (Reference)	0.983 (0.876, 1.102)	0.841 (0.746, 0.948)	0.844 (0.742, 0.959)	0.001
Model 2	1 (Reference)	0.982 (0.875, 1.102)	0.846 (0.750, 0.954)	0.852 (0.746, 0.973)	0.002
*CKD*					
Model 1	1 (Reference)	0.967 (0.866, 1.080)	0.830 (0.739, 0.931)	0.844 (0.745, 0.955)	0.001
Model 2	1 (Reference)	0.968 (0.866, 1.082)	0.836 (0.744, 0.939)	0.854 (0.751, 0.970)	0.001

Model 1: Adjusted for age, gender, education level, averaged monthly income, current smoker, current drinker, physical activity and body mass index.

Model 2: Adjusted for age, gender, education level, averaged monthly income, current smoker, current drinker, physical activity, body mass index, red meat (g/day), white meat (g/day), fish (g/day), egg (g/day), milk (g/day), vegetable (g/d) and fruit (g/d).

The results of the sensitivity analyses are revealed in Supplementary Tables S2 and S3. The relationships between quartiles of total beans intake with reduced eGFR, albuminuria and CKD prevalence in the total population remained almost unchanged when we reconducted the logistic analyses by additionally adjusting for T2DM, hypertension, dyslipidemia and hyperuricemia or adjusting dietary patterns instead of a single food item in model 2 (all *p* for linear trend <0.05). Only the inversed relationships of quartiles of total beans intake with albuminuria prevalence became nonsignificant in women when reconducting the logistic analyses by gender (Supplementary Table S2).

### Subgroup analyses

**Figure 3:**
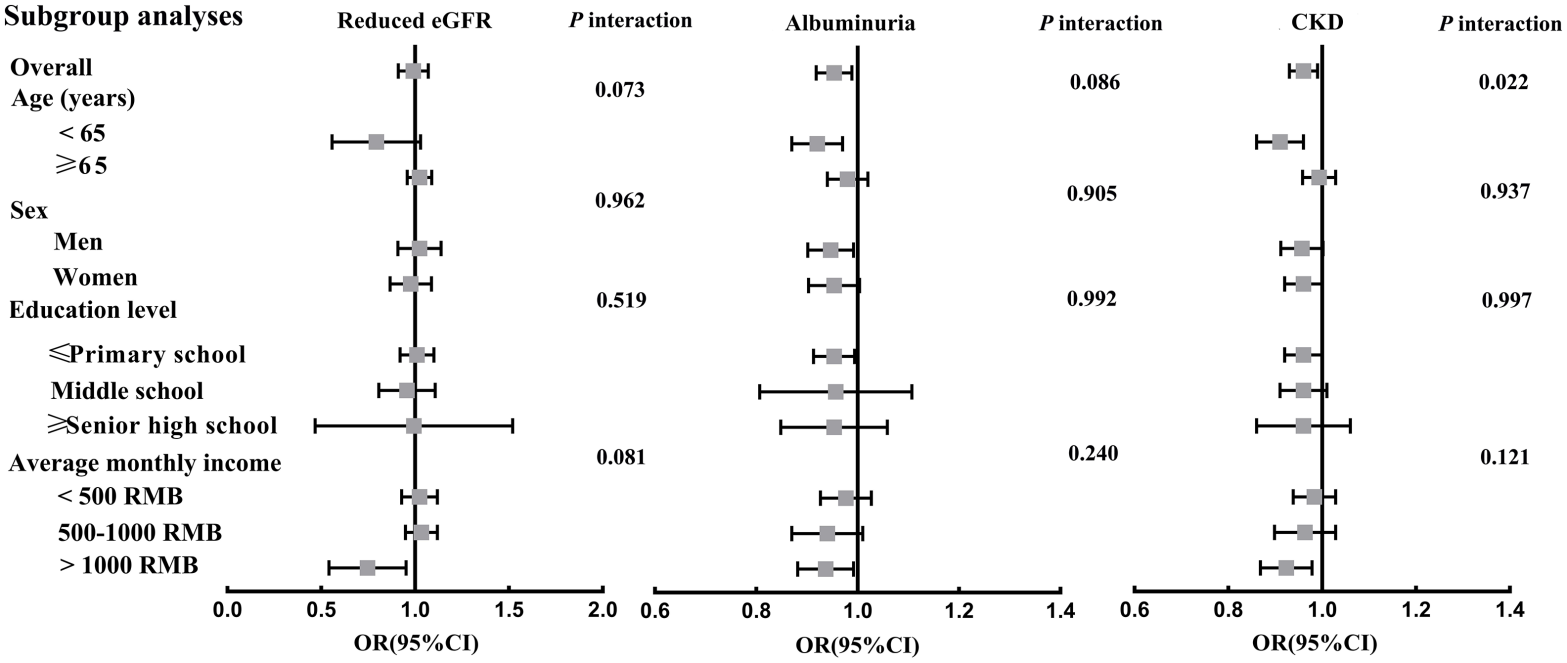
Associations between per 50 g/day increment in total beans intake and reduced eGFR, albuminuria and CKD prevalence in subgroup analyses. Adjusted for age, gender, education level, averaged monthly income, current smoker, current drinker, physical activity, body mass index, red meat (g/day), white meat (g/day), fish (g/day), egg (g/day), milk (g/day), vegetable (g/d) and fruit (g/d), T2DM, hypertension, dyslipidemia and hyperuricemia.

Figure 3, Supplementary Table S4 presented the relationships between per 50 g/day increment in total beans intake and reduced eGFR, albuminuria and CKD prevalence in each subgroup. Overall, per 50 g/day increment in total beans intake was significantly associated a 5 and 4% decreased prevalence of albuminuria and CKD, respectively, while not associated with reduced eGFR. However, the inverse relationships of total beans intake with albuminuria and CKD were only significant in the subgroups of men, age < 65 years, and average monthly income >1,000, and was borderline significant or nonsignificant in the other subgroups. In addition, only a significant interaction of total beans intake and age group on CKD risk was found (*p* = 0.022), in which the negative relationship of total beans intake with CKD was statistically significant in those aged <65 but not in those age ≥ 65 (Figure 3).

## Discussion

In the large-sample cross-sectional study, the main findings suggested that a higher beans intake was associated with a lower prevalence of albuminuria and CKD among rural adults, and linear trends were observed by increasing quartiles of total beans intake (all *p* for linear trend <0.05). In addition, these inverse relationships were significant in the subgroups of men, elder, and high-income participants, with a significant interactive effect of beans intake and age on CKD prevalence.

Soy foods are an excellent source of essential nutrients, including proteins, soluble dietary fibers, unsaturated fatty acids, iron, and isoflavones, constituting bioactive components (29). Many studies have found beneficial effects of soy or beans consumption on multiple chronic metabolic health outcomes, including hypertension, diabetes, hyperuricemia and dyslipidemia (9, 21, 30, 31), which are relevant risk factors of CKD. Nevertheless, few studies have investigated the direct relationship between soy food/beans intake and CKD risk, and no large population-based epidemiologic studies have been conducted. Based on our best knowledge, the present study is the first to estimate these relationships and find that total beans intake was independently associated with decreased prevalence of albuminuria and CKD among rural adults.

Although the evidence is limited, some relevant studies can support the current study’s findings. The potential kidney benefits of soy products have attracted widespread public health attention in recent years (32), because soy or beans are considered good representative sources of plant-based proteins (33). Some studies found that a high-quality plant-based diet can delay CKD progression, protect the endothelium, and decrease proteinuria (34, 35). A recent cohort study conducted in a sample of 14,686 adults found that higher adherence to a healthy plant-based diet was related to a lower risk of CKD and a slower eGFR decline (36). Moreover, in several nutritional studies, overall healthy dietary patterns that included rich in legumes or beans were associated with the risk of incident CKD in general populations or may reduce the risk of CKD progression or mortality (20, 37, 38). However, an independent health effect of beans intake on the outcome has not been shown.

Additionally, previous studies usually concluded that substituting soy protein for animal protein improved kidney function in animal models (19) or decreased glomerular hyperfiltration in human studies (18). However, there are also some inconsistent findings. Marion et al. have not observed beneficial effects when using soy protein instead of animal protein to attenuate proteinuria in the soy-treated group compared with the control (19). In addition, a meta-analysis involving 280 CKD patients suggested a protective effect of soy protein intake on SCR and serum phosphorus concentrations. Still, soy protein consumption did not affect the glomerular filtration rate (17). Similarly, our study also found higher beans intake was not significantly associated with a lower prevalence of reduced eGFR, but total beans intake is associated with decreased prevalence of albuminuria and CKD, which is comparable to the findings of most previous studies. These inconsistent findings may be due to the differences in the study region, design and sample size, and the types of beans intake and the approach to evaluating beans intake. More studies are needed to confirm our findings concerning the relationship between the total bean intake, as well as specific soy foods, and the risk of CKD based on a large population, in different countries.

The subgroup analyses showed that the inverse relationships of beans intake and albuminuria and CKD prevalence remained stable in the subgroups of men, age < 65 years, and average monthly income >1,000. Still, they became nonsignificant in women aged >65 years and lower income groups. The gender or age difference may be ascribed to the interaction effect of age and beans intake on CKD (*p* for interaction = 0.022). Our findings were similar in some ways to those of previous studies (39, 40), which found the health effect of soy foods and soy isoflavones only presented in younger and premenopausal women. It is speculated that the age difference may be due to a complex interaction between beans intake, estrogen, and estrogen receptor expression levels in women (41). Therefore, the nonsignificant results among women and older people in this study may be affected by estrogen levels in postmenopausal older women. There is still no plausible explanation for these observed differences, as the research is limited. More studies are warranted to detect possible causes for these differences.

The pathways mechanism beans intake to CKD are not yet clear, while some potential pathways can explain our findings to a certain extent. Soy or beans products contain high-quality protein, polyunsaturated fatty acids, carbohydrates, and fiber, as well as phytochemicals such as isoflavones, phytosterols, and lecithin (17, 29). These components may reduce inflammation, oxidative stress, and endothelial dysfunction and confer unique health benefits (36, 42). A recent animal study also found that dietary fermented soy extract may alleviate CKD in a mouse model of adenine-induced CKD *via* inhibiting inflammation and modulation of gut microbiota (43). In addition, soy or beans consumption has been suggested to be related to multiple risk factors of CKD, such as hypertension, diabetes, dyslipidemia and hyperuricemia, which may further contribute to the development of CKD. Nevertheless, the negative relationships between total beans intake with reduced eGFR, albuminuria and CKD prevalence remained stable after adjusting for these disease risk factors in sensitivity analyses (Supplementary Table S2). Hence, daily total bean intake may effectively inhibit chronic inflammation and slow kidney disease progression.

### Strengths and limitations

The current study is the first to evaluate the effects of beans intake on renal function and CKD prevalence among rural adults. The strengths include a large sample-based population study from the latest survey (2018–2022), the use of a clinically relevant definition of CKD, a validated FFQ for dietary, and a series of sensitivity analyses and subgroup analyses, which allowed for sufficient statistical power to confirm the robustness of our results.

Several limitations in the study need to be acknowledged. First, because of the cross-sectional design, the causal relationships between total beans intake with renal function and CKD prevalence cannot be inferred. Further prospective studies are needed to validate the current findings. Second, dietary data were self-reported with inevitable measurement errors. However, the FFQ used had been validated as an alternative to the 3-day-24-h dietary survey method to evaluate the diet of a large rural population (23). Third, the FFQ contained 13 items and was designed to assess the mean quantity and frequency of each item consumed over the previous year. Collecting the accurate amount of each type of bean intake is hard. Therefore, we could not determine which kinds of beans were consumed, and the detailed nutrient components played a major role in delaying the progression of CKD. More detailed categorization of food items and expansion of foods within each food group would be helpful in future studies. Fourth, although many potential risk factors of CKD were considered in these analyses, some residual confounding factors were not collected, such as medication use of chronic nephrotoxic drugs and digestive diseases drugs, which affected the absorption of soy products. Finally, the study was conducted among rural populations; therefore, the generalization of the present findings to other countries or populations is uncertain. Nevertheless, this is the first study to show an inverse relationship between total beans intake and CKD prevalence in China, providing some new public health guidance for preventing CKD.

## Conclusion

In conclusion, beans intake was independently associated with decreased prevalence of albuminuria and CKD among rural adults. These inverse relationships were significant in the subgroups of men, elder, and high-income participants, with a significant interactive effect of beans intake and age on CKD prevalence. The findings highlight the beneficial effect of beans food on renal function and provide important evidence for public health and clinical practices that promoting the consumption of soy or beans products may be an effective strategy for preventing renal damage and CKD development. To confirm these findings, more large-scale prospective studies and randomized controlled trials are needed.

## Data availability statement

The raw data supporting the conclusions of this article will be made available by the authors, without undue reservation.

## Ethics statement

The studies involving human participants were reviewed and approved by Zhengzhou University Medical Life Science Committee (Ethic approval code: [2015] MEC (S128)). The patients/participants provided their written informed consent to participate in this study.

## Author contributions

ZL and CW conceived and designed study. WL, XL, ZZ, and DL coordinated data collection. LY and XD conducted the analyses and wrote the manuscript. All authors contributed to the article and approved the submitted version.

## Funding

This research was supported by the Chinese Nutrition Society-Danone Dietary Nutrition Research and Education Fund (grant no: DIC2020-04) and Yum China Dietary Health Foundation (grant no: CNS-YUM2020A19), Science and Technology Innovation Team Support Plan of Colleges and Universities in Henan Province (grant no: 21IRTSTHN029), Key Research Program of Colleges and Universities in Henan Province (grant no: 21A330007) Foundation of National Key Program of Research and Development of China (grant no: 2016YFC0900803), The funders had no role in the study design, data collection and analysis, decision to publish, or preparation of the manuscript.

## Conflict of interest

The authors declare that the research was conducted in the absence of any commercial or financial relationships that could be construed as a potential conflict of interest.

## Publisher’s note

All claims expressed in this article are solely those of the authors and do not necessarily represent those of their affiliated organizations, or those of the publisher, the editors and the reviewers. Any product that may be evaluated in this article, or claim that may be made by its manufacturer, is not guaranteed or endorsed by the publisher.

## Clinical trial registration

Before our study the Henan Rural Cohort Study has been registered in the Chinese Clinical Trial Register (Registration number: ChiCTR-OOC-15006699).
