# Supplementary material for: Relationships of beans intake with chronic kidney disease in rural adults: A large-scale cross-sectional study
Source: Front Nutr. 2023 Apr 4;10:1117517. doi: 10.3389/fnut.2023.1117517 (PMC10111024; doi:10.3389/fnut.2023.1117517)
Supplement: Supplementary file 1 [file Data_Sheet_1.docx]

Supplementary Material

**Table S1** Basic characteristics of study participants according to indicators of kidney injury.

**Table S2** Relationships between quartiles of total beans intake with reduced eGFR, albuminuria and CKD risk in sensitivity analyses by gender.

**Table S3** Relationships between quartiles of total beans intake with reduced eGFR, albuminuria and CKD risk in sensitivity analyses.

**Table S4** Relationships between per 50g/day increment in total beans intake and reduced eGFR, albuminuria and CKD risk in subgroup analyses.

**Figure S1** Flowchart for participant inclusion.

**Table S1. Basic characteristics of study participants according to indicators of kidney injury.**

| Variables | Participants with  reduced eGFR  (N = 366) | | Participants with  albuminuria  (N = 2448) | Participants without  kidney injury (N = 18057) | |
| --- | --- | --- | --- | --- | --- |
| Age (year, mean ± SD) | 69.96 ± 8.95 |  | 63.41 ± 10.82 | | 59.62 ± 11.30 |
| Gender (n, %) |  |  |  | |  |
| Men | 131 (35.79) |  | 874 (35.70) | | 6881 (38.11) |
| Women | 235 (64.21) |  | 1574 (64.29) | | 11176 (61.89) |
| Education level (n, %) |  |  |  | |  |
| ≤Primary school | 267 (72.95) |  | 1420 (58.10) | | 8561 (47.50) |
| Middle school | 86 (23.50) |  | 811 (33.18) | | 7055 (39.14) |
| ≥Senior high school | 13 (3.55) |  | 213 (8.72) | | 2407 (13.36) |
| Average monthly income (n, %) |  |  |  | |  |
| <500 RMB | 183 (50.00) |  | 1024 (41.83) | | 5978 (33.11) |
| 500-1000 RMB | 82 (22.40) |  | 615 (25.12) | | 4853 (26.88) |
| >1000 RMB | 101 (27.60) |  | 809 (33.05) | | 7226 (40.01) |
| Current smoker, n (%) | 40 (10.93) |  | 342 (14.97) | | 3052 (16.90) |
| Current drinker, n (%) | 29 (7.92) |  | 303 (12.38) | | 2775 (15.36) |
| Physical activity, n (%) |  |  |  | |  |
| Light | 195 (53.28) |  | 1147 (46.85) | | 7120 (39.43) |
| Moderate | 109 (29.78) |  | 782 (31.94) | | 6113 (33.85) |
| Vigorous | 62 (16.94) |  | 519 (21.20) | | 4824 (26.72) |
| BMI (kg/m^2^), mean ± SD | 24.58±3.50 |  | 25.19±3.76 | | 24.50±3.45 |
| Red-meat (g/day), mean ± SD | 27.96±47.42 |  | 32.90±59.11 | | 38.59±74.74 |
| White-meat (g/day), mean ± SD | 16.07±35.60 |  | 20.20±81.91 | | 21.23±54.65 |
| Fish (g/day), mean ± SD | 4.50±13.62 |  | 6.50±31.54 | | 7.91±32.35 |
| Egg (g/day), mean ± SD | 43.22±39.19 |  | 47.02±39.76 | | 48.27±40.51 |
| Milk (g/day), mean ± SD | 53.89±98.58 |  | 54.06±100.33 | | 48.57±90.56 |
| Vegetable intake (g/d), mean ± SD | 356.56±238.40 |  | 366.28±229.07 | | 368.65±221.86 |
| Fruit intake (g/d), mean ± SD | 124.27±147.73 |  | 129.66±146.42 | | 150.59±170.11 |
| Beans (g/day), mean ± SD | 36.91±72.47 |  | 37.46±68.77 | | 42.82±81.65 |
| Hypertension, n (%) | 218 (59.56) |  | 1378 (56.29) | | 4946 (27.40) |
| T2DM, n (%) | 83 (22.74) |  | 677 (27.73) | | 2094 (11.61) |
| Dyslipidemia, n (%) | 248 (67.76) |  | 1674 (68.41) | | 10697 (59.26) |
| Hyperuricemia, n (%) | 214 (58.47) |  | 489 (19.98) | | 2254 (14.14) |
| eGFR (mL/min/1.73 m^2^), mean ± SD | 47.81±11.32 |  | 97.34±23.49 | | 102.44±17.30 |
| ACR (mg/g), mean ± SD | 127.28±356.68 |  | 128.96±272.29 | | 9.18±6.21 |

SD, standard deviation; BMI, body mass index; eGFR, estimated glomerular filtration rate; ACR, urinary albumin to creatinine ratio.

**Table S2** **Relationships between quartiles of total beans intake with reduced eGFR, albuminuria and CKD prevalence in sensitivity analyses by gender.**

| **Outcome** | **Total beans intake (g/day)** | | | | ***P* for linear trend** |
| --- | --- | --- | --- | --- | --- |
|  | **Q1** | **Q2** | **Q3** | **Q4** |  |
| **Total population** | |  |  |  |  |
| **Reduced eGFR** | |  |  |  |  |
| Model 2* | **1 (Reference)** | 0.819 (0.617, 1.086) | 0.743 (0.549, 1.005) | 0.739 (0.531, 1.030) | 0.038 |
| **Albuminuria** |  |  |  |  |  |
| Model 2* | **1 (Reference)** | 0.965 (0.857, 1.086) | 0.820 (0.725, 0.928) | 0.824 (0.719, 0.943) | <0.001 |
| **CKD** |  |  |  |  |  |
| Model 2* | **1 (Reference)** | 0.952 (0.849, 1.067) | 0.810 (0.718, 0.913) | 0.824 (0.723, 0.939) | <0.001 |
| **Men** |  |  |  |  |  |
| **Reduced eGFR** | |  |  |  |  |
| Model 2* | **1 (Reference)** | 0.642 (0.394, 1.045) | 0.564 (0.336, 0.945) | 0.743 (0.436, 1.266) | 0.143 |
| **Albuminuria** |  |  |  |  |  |
| Model 2* | **1 (Reference)** | 0.880 (0.716, 1.081) | 0.721 (0.584, 0.891) | 0.764 (0.611, 0.954) | 0.004 |
| **CKD** |  |  |  |  |  |
| Model 2* | **1 (Reference)** | 0.857 (0.702, 1.046) | 0.713 (0.581, 0.875) | 0.779 (0.629, 0.966) | 0.005 |
| **Women** |  |  |  |  |  |
| **Reduced eGFR** | |  |  |  |  |
| Model 2* | **1 (Reference)** | 0.910 (0.641, 1.290) | 0.834 (0.573, 1.213) | 0.712 (0.464, 1.091) | 0.106 |
| **Albuminuria** |  |  |  |  |  |
| Model 2* | **1 (Reference)** | 1.005 (0.869, 1.162) | 0.878 (0.753, 1.023) | 0.853 (0.718, 1.013) | 0.024 |
| **CKD** |  |  |  |  |  |
| Model 2* | **1 (Reference)** | 0.999 (0.868, 1.149) | 0.866 (0.747, 1.004) | 0.842 (0.713, 0.995) | 0.012 |

Model 2*: Adjusted for age, gender (only for total population), education level, averaged monthly income, current smoker, current drinker, physical activity, body mass index, red-meat (g/day), white-meat (g/day), fish (g/day), egg (g/day), milk (g/day), vegetable (g/d) and fruit (g/d), T2DM, hypertension, dyslipidemia and hyperuricemia.

**Table S3** **Relationships between quartiles of total beans intake with reduced eGFR, albuminuria and CKD prevalence in sensitivity analyses.**

| **Outcome** | **Total beans intake (g/day)** | | | | ***P* for linear trend** |
| --- | --- | --- | --- | --- | --- |
|  | **Q1** | **Q2** | **Q3** | **Q4** |  |
| **Reduced eGFR** |  |  |  |  |  |
| Model 2 | **1 (Reference)** | 0.810 (0.616, 1.066) | 0.733 (0.548, 0.981) | 0.754 (0.550, 1.033) | 0.037 |
| **Albuminuria** |  |  |  |  |  |
| Model 2 | **1 (Reference)** | 0.977 (0.870, 1.096) | 0.836 (0.744, 0.946) | 0.828 (0.727, 0.943) | <0.001 |
| **CKD** |  |  |  |  |  |
| Model 2 | **1 (Reference)** | 0.962 (0.861, 1.075) | 0.836 (0.737, 0.926) | 0.826 (0.729, 0.936) | <0.001 |

Model 2: Adjusted for age, gender, education level, averaged monthly income, current smoker, current drinker, physical activity, body mass index and dietary pattern (instead of a single food item, the four-cluster dietary patterns were obtained by factor analysis using the standard principal component analysis method, dietary pattern I with a high intake of red meat, white meat and fish; pattern II with a high intake of milk and eggs; pattern III with a high intake of grains, nuts and beans; and pattern IV with a high intake of vegetables, staple food, and fruits)

**Table S4** Relationships between per 50g/day increment in total beans intake and reduced eGFR, albuminuria and CKD prevalence in subgroup analyses.

|  | ***OR* (95% *CI*) for Reduced eGFR** | ***P* _interaction_** | ***OR* (95% *CI*) for** **Albuminuria** | ***P* _interaction_** | | ***OR* (95% *CI*) for CKD** | ***P* _interaction_** |
| --- | --- | --- | --- | --- | --- | --- | --- |
| **Overall** | 0.99 (0.91, 1.07) |  | 0.95 (0.92, 0.99) | |  | 0.96 (0.93, 0.99) |  |
| **Age (years)** |  | 0.073 |  | | 0.086 |  | 0.022 |
| <65 | 0.77 (0.52, 1.04) |  | 0.92 (0.87, 0.97) | |  | 0.91 (0.86, 0.96) |  |
| ≥65 | 1.02 (0.96, 1.09) |  | 0.98 (0.94, 1.02) | |  | 0.99 (0.96, 1.03) |  |
| **Sex** |  | 0.962 |  | | 0.905 |  | 0.937 |
| Men | 1.02 (0.91, 1.14) |  | 0.95 (0.90, 0.99) | |  | 0.96 (0.91, 0.99) |  |
| Women | 0.97 (0.87, 1.09) |  | 0.96 (0.92, 1.00) | |  | 0.96 (0.92, 1.00) |  |
| **Education level** |  | 0.519 |  | | 0.992 |  | 0.997 |
| ≤Primary school | 1.01 (0.92, 1.10) |  | 0.96 (0.91, 1.00) | |  | 0.96 (0.92, 1.00) |  |
| Middle school | 0.95 (0.81, 1.11) |  | 0.96 (0.91, 1.01) | |  | 0.96 (0.91, 1.01) |  |
| ≥Senior high school | 0.90 (0.52, 1.56) |  | 0.95 (0.85, 1.06) | |  | 0.96 (0.86, 1.06) |  |
| **Average monthly income** |  | 0.081 |  | | 0.240 |  | 0.121 |
| <500 RMB | 1.02 (0.93, 1.12) |  | 0.97 (0.93, 1.03) | |  | 0.98 (0.94, 1.03) |  |
| 500-1000 RMB | 1.03 (0.95, 1.12) |  | 0.94 (0.87, 1.01) | |  | 0.96 (0.90, 1.03) |  |
| >1000 RMB | 0.73(0.55, 0.96) |  | 0.94 (0.88, 0.99) | |  | 0.92 (0.87, 0.98) |  |

Adjusted for age, gender, education level, averaged monthly income, current smoker, current drinker, physical activity, body mass index, red-meat (g/day), white-meat (g/day), fish (g/day), egg (g/day), milk (g/day), vegetable (g/d) and fruit (g/d), T2DM, hypertension, dyslipidemia and hyperuricemia.

Five rural counties (Tongxu, Suiping, Yima, Xinxiang and Yuzhou counties) in Henan Province of China were selected

21,079 participants completed the routine blood measurements

A total of 28,630 rural residents aged 18-79 years completed the questionnaires and physical examination

135 participants with missing data on beans intake (N=15), eGFR (N=4) and ACR (N=116) were further excluded

20,733 participants were finally included in the current analysis

211 participants were excluded due to diagnosed cancers (N=200) and Kidney failure (N=11)

**Figure S1.** Flowchart for participant inclusion
